# Heterologous Immunity: Role in Natural and Vaccine-Induced Resistance to Infections

**DOI:** 10.3389/fimmu.2019.02631

**Published:** 2019-11-08

**Authors:** Babita Agrawal

**Affiliations:** Department of Surgery, Faculty of Medicine and Dentistry, University of Alberta, Edmonton, AB, Canada

**Keywords:** heterologous (non-specific) effects of vaccines, heterologous immunity, T cells, antibody, innate and adaptive immune response

## Abstract

The central paradigm of vaccination is to generate resistance to infection by a specific pathogen when the vacinee is re-exposed to that pathogen. This paradigm is based on two fundamental characteristics of the adaptive immune system, specificity and memory. These characteristics come from the clonal specificity of T and B cells and the long-term survival of previously-encountered memory cells which can rapidly and specifically expand upon re-exposure to the same specific antigen. However, there is an increasing awareness of the concept, as well as experimental documentation of, heterologous immunity and cross-reactivity of adaptive immune lymphocytes in protection from infection. This awareness is supported by a number of human epidemiological studies in vaccine recipients and/or individuals naturally-resistant to certain infections, as well as studies in mouse models of infections, and indeed theoretical considerations regarding the disproportional repertoire of available T and B cell clonotypes compared to antigenic epitopes found on pathogens. Heterologous immunity can broaden the protective outcomes of vaccinations, and natural resistance to infections. Besides exogenous microbes/pathogens and/or vaccines, endogenous microbiota can also impact the outcomes of an infection and/or vaccination through heterologous immunity. Moreover, utilization of viral and/or bacterial vaccine vectors, capable of inducing heterologous immunity may also influence the natural course of many infections/diseases. This review article will briefly discuss these implications and redress the central dogma of specificity in the immune system.

## Introduction

Studies in humans and mouse models have clearly demonstrated that exposure or infection with one pathogen can induce and/or modify the immune response against another unrelated pathogen. This is what's defined as heterologous immunity (1, 2). The ability of an individual to respond to a pathogen is influenced by its exposure history to a significant extent, both by pathogenic microbes and commensals (microbiota) (2). Heterologous immunity could boost or weaken protective immunity against a pathogen, and/or induce severe immunopathology or tolerance against self-antigens. Therefore, there must be a delicate balance between protective immunity and immunopathology, and heterologous immunity can play an important role in tilting this balance. Heterologous adaptive immunity was initially thought to be due to high levels of amino acid sequence similarities in T cell and B cell epitopes among antigens of different pathogens, but has now broadened in scope with the realization of the highly cross-reactive nature of adaptive immune lymphocytes (3). With the expanding understanding of heterologous immunity, there is a need to re-think and re-examine its impact on vaccines, resistance to infections, protective vs. detrimental immunity, as well as autoimmunity. In this article, I will briefly review heterologous immunity, and its impact on natural immunity to infections, vaccine vectors and vaccine-mediated protection by describing, (1) the realm of cross-reactive adaptive lymphocytes, (2) evidence of cross-reactivity from vaccine studies, (3) animal and human model experiments to demonstrate cross-reactivity between a broad range of pathogens, (4) possible influence of cross-reactivity on natural resistance to infections, (5) role of microbiota in heterologous immunity, (6) vaccine vectors and heterologous immunity, and (7) discussion and future prospects. In addition to cross-reactive lymphocytes, a network of cytokines, regulatory cells and trained innate immune cells contribute to manifesting heterologous immunity (4, 5). However, this article is more focused on cross-reactive adaptive immune lymphocytes.

## The Realm of Cross-reactive Adaptive Cellular Immunity

**Figure 1:**
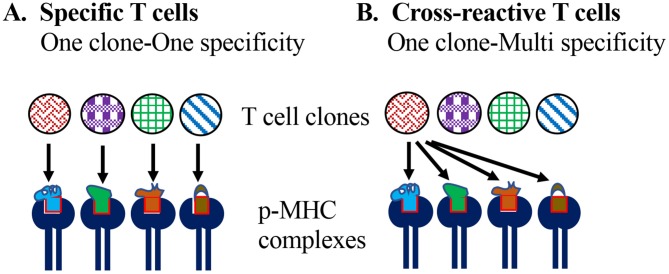
Specificity vs. cross-reactivity of T cells. **(A)** According to the one-clone-one-specificity model, individual clones of T cells recognize one specific peptide epitope in the context of self MHC molecules, and do not recognize or get stimulated with any other peptide. **(B)** Model of T cell cross-reactivity implies an individual T cell clone can recognize multiple peptide epitopes in the context of the self MHC molecule.

Heterologous immunity is the induction of an immune response to an unrelated pathogen/antigen upon exposure to a different pathogen/antigen. Conceptually, cross-reactivity (or poly-specificity) of lymphocytes in antigen (or epitope) recognition is foundational to heterologous adaptive immunity (Figure 1).

The essence of clonal selection theory of T cells is based on: one epitope specificity-one T cell clonotype, which defines and contributes to the high specificity of adaptive cellular immune responses and differentiates them from innate lymphocytes that rely on broad pattern recognition (6, 7). Accordingly, it is assumed that T cells bearing a T cell receptor (TCR) for a specific peptide epitope [~9 amino acids (aa) for CD8 and ~11 aa for CD4] emerge in an individual long before they are exposed to the corresponding foreign antigen through random variable (V), diversity (D) and joining (J) (VDJ) regions' recombination of TCR α and β chains and permutations of α and β chain heterodimers, as well as rounds of positive and negative selection in the thymus.

The high specificity and the apparent stringency of the specific interaction of peptide bound with major histocompatibility complex and T cell receptor (p-MHC:TCR) complexes were demonstrated by X-Ray crystallography studies (8). These structural studies were followed by a wide range of flow cytometry studies encompassing p-MHC tetramer-based detection of T cells with stringent peptide specificity, supporting and perpetuating the one specificity-one clonotype theory (9). Observations contradicting this theory were few and often not well-documented in literature. Technical limitations in clearly defining and characterizing cross-reactivity (or heterologous immunity) at the molecular level and reliance on empirical cellular methodologies could partially explain an apparent scarcity of reporting of cross-reactivity in experimental systems. Regardless, they have substantial clinical implications in vaccine and immunotherapy applications.

It has been predicted that high affinity would reflect a better quality immune response, and indeed, in viral immunology the demonstration of specific p-MHC binding T cells was suggested to correlate to protection from infection or viral clearance (10, 11). Nevertheless, the relationship between the affinity of the TCR and p-MHC, and the subsequent immune response is not direct and may lead to unexpected immune responses and undesired consequences. Cancer immunotherapy has anticipated that an enhanced therapeutic effect might occur when TCR affinity is increased in TCR-based therapeutics such as chimeric antigen receptors bearing T cells (CAR-T) cells and T cell adoptive transfer. However, clinical trials using high affinity T cell adoptive transfer against the melanoma associated antigen-3 (MAGE-3), resulted in off-target cross-reactivity against a cardiac-associated Titin antigen and fatality (12). There was only 55% homology between the MAGE-3 peptide and Titin-derived peptide, but X-ray studies demonstrated similarity in p-MHC complexes, which formed the basis for the observed cross-reactivity (13, 14). Thus, TCR-pMHC affinity alone may not predict the efficiency of the immune response or clinical success in both vaccine and T-cell adoptive transfer based therapeutics.

Physiologically, it is rather puzzling that the T cells are believed to be individually highly specific, and yet expected to collectively respond to a huge number of foreign antigens in a host to provide protective immunity against a vast number of pathogens of various classes during lifelong exposures. Also, a logistic problem that exists in the available functional T cell repertoire in an individual is the limited number of T cell clonotypes, i.e., ~10^6^ in mice and ~10^8^ in humans, which would severely limit the extent of immune responses generated, especially since at least an estimated >10^15^ peptides must be effectively responded against, in a host's lifetime (3, 15, 16). Based on mathematical calculation of this disparity, it has been hypothesized that each T cell clonotype needs to recognize ~10^6^ different p-MHC combinations in order for adaptive cellular immune system to be effective (3, 17). Therefore, revisiting of the concept of clonal selection and one-specificity-one T cell is warranted. Classical CD8^+^ and CD4^+^ T cells recognize peptide epitopes of 9–16 aa in the context of MHC class I and class II molecules, respectively. However, in their recognition, the TCR only contacts 3–5 aa of the peptide bound to the MHC molecules, and at minimum, 4 aa long peptides have been shown to stimulate T cell activation (15, 18). TCR recognizing a p-MHC complex glides over a relatively flat surface, culminating with favorable interaction between the complementarity determining region (CDR) of the TCR and 3–4 amino acids of the MHC bound peptide. Thereafter, the TCR binds with the p-MHC complex generally with short range weak intermolecular bonds, i.e., van der Waal's forces (2). TCR:p-MHC binding is characterized with low numbers of hydrogen bonds and almost no covalent bonds, resulting in low affinities of interaction (19). Multimerization of p-MHC provides the strength of a T cell recognizing p-MHC on antigen presenting cells (APCs), that results in activation thresholds (20). In addition to the low-affinity binding, the complementarity determining region (CDR) of the TCR demonstrates structural rearrangements and plasticity in conformation in binding to the p-MHC complex, as well as variations in angles of docking onto p-MHC, and conformational shifts in both peptide and MHC of the p-MHC complex. All these changes result in thermodynamic and physicochemical mechanisms for cross-reactivity of the TCR (15). Furthermore, structural degeneracy of amino acids in the peptide bound to MHC supports the cross-reactive recognition by the TCR (15). These include the physicochemical properties of the amino acids that provide the hydrophobic characteristics for Van der Waal's interaction between TCR and p-MHC instead of the strict identity of the amino acid. These mechanisms, along with the minimal contact requirement of the TCR on p-MHC, i.e., 3–5 aa, would provide conditions conducive for cross-reactivity. In addition, T cells are positively selected in the thymus based on a limited selection of self-peptide epitopes bound to MHC molecules, and yet result in T cells recognizing a wide variety of previously unseen foreign peptides. This selection process, in addition to the structural considerations mentioned earlier, add to an absolute inherent requirement for T cells to be cross-reactive (21). Based on these features, it is understandably a very difficult task to recognize cross-reactivity using current molecular techniques. Most characterization of cross-reactivity of T cells has been achieved through empirical observations in humans and animal models, using epidemiological studies and cellular techniques.

## Vaccines and Evidence (or Manifestation) of Heterologous Immunity

Some of the most common and longest-used vaccines in humans are Vaccinia (smallpox vaccine, for smallpox virus), BCG (Bacille Calmette Guerin, for tuberculosis), Measles (for measles virus), OPV (oral polio vaccine), and DTP (for diphtheria, tetanus, and pertussis) (22, 23) (https://www.cdc.gov/vaccines/vpd/index.html). The human use of these vaccines has been very effective in preventing infection with the corresponding pathogen and associated mortality, and has even led to the eradication of smallpox virus infections from the world and polio to the verge of eradication. In addition to the specific effect of these vaccines in preventing the targeted infection, the non-specific or heterologous effect of these vaccines in preventing infections with unrelated pathogens has been recognized through epidemiological studies in human populations (22, 24–27) as described below.

Worldwide mandatory smallpox vaccination during the 1960s and 70s, contributed to the disease being eliminated in 1980 (https://www.cdc.gov/smallpox/history/history.html). Anecdotally, scientists in the nineteenth and early twentieth century reported positive effects of small pox vaccine in multiple diseases other than small pox such as papillomas, chronic skin disorders, eyes, ear, nose and throat disorders, measles, scarlet fever, whooping cough, and syphilis (28). From a cohort of 3,559 individuals in Denmark, it has been found that smallpox vaccination is associated with a reduced risk of infectious disease hospitalization in a high-income setting (29). There has also been some preliminary suggestion that prior immunization with small pox vaccine may provide an individual with some degree of protection to subsequent Human Immunodeficiency Virus (HIV) infection and that the worldwide termination of smallpox vaccination in 1980 may have partially allowed the HIV epidemic to explode (30). It has been shown in epidemiological studies from Guinea-Bissau and Denmark that smallpox and/or BCG vaccination is associated with a reduced risk of HIV-1 infection in women (27). Using CCR5^+^ T cells from unvaccinated or smallpox-vaccinated individuals, it was shown that HIV-1 replicated to lower levels in cells from vaccinated individuals compared to unvaccinated people (30). In another study using 97 women with or without a smallpox-vaccine scar, however, CCR5 expression in T cells could not be correlated to protection from HIV-1 infection with vaccination status (31), suggesting that modulation of CCR5 expression apparently does not contribute to the observed protection. It remains to be seen whether heterologous immunity contributed to an apparent protection from HIV-1 infection upon smallpox and/or BCG vaccination, even to a small extent. Overall, these reports suggest a non-specific protective effect of smallpox vaccine on a number of infectious diseases in human population, the mechanisms of which are not clear yet.

BCG vaccination in infants has been shown to reduce infant mortality due to childhood infections such as respiratory infections and sepsis unrelated to tuberculosis in high mortality settings as well as in USA and Europe (24, 32–34). Immunization with BCG has also been shown to reduce the incidence of allergic diseases, and autoimmune/inflammatory diseases such as type I diabetes (T1D) and multiple sclerosis (MS) (35). Removal of the infant BCG vaccination program due to decline in TB cases has been shown to be correlated with increased incidence of respiratory infections, melanoma, lymphoma, atopic dermatitis, asthma etc. (36). Intra-vesicular treatment with BCG has become the mainstream treatment of bladder cancer (37). Along the same lines, studies in animal models have shown that BCG immunization of mice leads to prevention in development of type 1 diabetes (T1D) as well as resistance to vaccinia virus infection (38, 39). Detailed mechanisms of these effects of BCG vaccination are still unclear. However, animal model studies have shown a role of CD4^+^ T cells, as well as trained innate immunity (39). Role of trained innate immunity in providing heterologous immunity upon BCG vaccination has also been demonstrated in humans (26, 40, 41).

Measles vaccine given to infants has been shown to reduce childhood mortality by infections other than measles by 30–86% in 10 different cohort studies from different countries (42). Similarly, in a study encompassing the years 2002–2014, oral polio vaccine, used widely to eradicate polio has been shown to reduce mortality by 19% (range 5–32%) in children <5 years of age independent of its effect on polio (43). Furthermore, in a randomized clinical trial, vaccination with OPV and BCG at birth demonstrated 32% (0–57%) lower infant mortality than BCG alone (44).

The diphtheria-tetanus-pertussis (DTP) vaccine shows excellent protection against the three targeted diseases, however, it has been shown that mortality in females (but not males) increases from other infectious diseases (45, 46). However, the increased female mortality was only found in children who had received DTP after measles vaccine producing high titers, whereas in subjects receiving measles and/or BCG vaccine after DTP, mortality rate declined substantially (45). It was suggested that immunization with DTP may deregulate the female immune system so that subsequent unrelated infections are fought inefficiently, whereas immunization with BCG or measles vaccine subsequent to DTP may circumvent the harmful effect of DTP (45). Therefore, the non-specific effects of vaccines must be thoroughly studied with respect to sex differences.

Potential mechanisms for heterologous effects of vaccines may include cross-reactivity between shared epitopes of unrelated pathogens, trained immunity in innate cells such as natural killer (NK), natural killer T cells (NKT) and monocytes, modulation of type 1, type 17, regulatory and memory T cells, cytokine responses, and modulation of mean concentration of antibodies as well as cross-reactive antibodies (47). Nevertheless, the exact contribution of each of these potential mechanisms in producing the observed heterologous effects of vaccines remains to be delineated.

## Heterologous Immunity Across a Broad Range of Pathogens

Heterologous immunity has been shown commonly among closely related pathogens, e.g., different subtypes of influenza A viruses and Dengue viruses, different members of the same family such as within flaviviruses and picornaviruses, and among unrelated pathogens including parasites, protozoa, bacteria, and viruses. It has been suggested that the history of exposure to various microbial infections and the resulting changes in the memory T cell repertoires determine the existence of a cross-reactivity network in each individual, and therefore cross-reactivity against multiple epitopes may be observed in an individual (48).

Heterologous immunity has been experimentally shown in mice by Welsh and Selin, who have reported that some levels of protection against vaccinia virus (VV) infection is obtained in mice that have been earlier exposed to infections with lymphocytic choriomeningitis virus (LCMV), murine cytomegalovirus (MCMV), Pichinde virus (PV), or influenza A virus (IAV) (1, 17, 49, 50). Furthermore, LCMV, PV, and MCMV all provide reciprocal cross-reactive immunity in mice (50). Interestingly, influenza virus infection provides cross-protective immunity against VV, but exacerbates infection with LCMV and MCMV. However, VV infection does not cross-protect against any of the tested heterologous pathogens and does not show reciprocal heterologous immunity (51). Our studies have demonstrated unexpected cross-reactivity between adenoviruses and Hepatitis C virus (52). Most of these examples of heterologous immunity have demonstrated a role for cross-reactive T cells, but other mechanisms may also be contributing toward the overall effect. With the induction of cross-reactive T cells, besides enhanced or inhibited clearance of a virus, T cell immunodominance patterns may be altered resulting in unusual skewing of T cell repertoires (53). It has been shown that heterologous virus challenge may lead to expansion of cross-reactive narrowly focused T cell repertoire and viral escape, whereas homologous viral challenge may allow expansion of more oligoclonal T cell responses (17, 54). It has also been suggested that memory T cells generated after an infection have a lower activation threshold and may be activated by the bystander effect local cytokines independent of TCR signaling, contributing significantly to heterologous immunity (55). Further, it must be highlighted that in most instances, heterologous immunity is not nearly as effective as specific immunity but may be sufficient to alter the otherwise severe course of a heterologous infection and mortality. The widespread overlap in heterologous immunity between these different viruses suggests that cross-reactivity among pathogens is prevalent.

A recent article demonstrated that sequential challenges of mice with Dengue virus (DENV), Yellow Fever virus (YFV), and Japanese Encephalitis virus (JEV), all members of flaviviruses, results in the induction of heterologous cellular and humoral immunity (56). Prior exposure to YFV and JEV produced high titer antibodies against DENV1, whereas prior exposure to DENV1 produced cross-reactive antibodies against JEV but not YFV (56). Interestingly, JEV and JFV primed mice demonstrated T cell cross-reactivity with each other, whereas DENV1 priming induced cross-reactive T cells against JEV but not YFV, paralleling the cross-reactivity demonstrated by antibodies. It was further demonstrated that humans also have cross-reactive T cells and antibodies similar to data obtained in mice. Overall, the results demonstrated that cross-reactive flavivirus immunity can provide enhanced protection to a heterologous infection.

There are also multiple examples of heterologous immunity between bacteria and viruses. Herpes virus infection in mice with MCMV and Murine c-Herpesvirus, has been demonstrated to induce protective immunity against bacterial pathogens such as *Listeria monocytogenes* and *Yersinia pestis* (57). It has been suggested that increased levels of IFN-γ induces activation in macrophages resulting in enhanced clearance of intracellular bacteria. Besides epidemiological studies suggesting induction of heterologous immunity upon BCG immunization as described in earlier section, BCG has also been shown to provide protective immunity to VV in mice, and this protection appears to be dependent on cross-reactive CD4^+^ T-cells (38). In contrast, BCG does not provide cross-reactive immunity against LCMV or MCMV in mice (38). A number of other heterologous effects of BCG appear to be related to its effect on innate immunity (58) and are not described here.

It has been demonstrated that a fungal species *Candida albicans* hyphal wall protein (Hyr1p) shares significant structural homology to a bacteria species *Acinetobacter baumannii* cell surface protein, and active (with rHyr1p) or passive (with anti-Hyr1p antibodies) immunization of mice protects them from systemic infection with *A. baumannii* and pneumonia (59). The observed cross-reactive/heterologous immunity among fungal and bacterial antigens was likely due to highly conserved B cell epitopes and 3-D structural homology between them. Most experimental studies of heterologous immunity have used animals (mice) immunized or challenged with a pathogen followed by determining the immune response or protection against an unrelated organism.

Heterologous immunity is rather difficult to demonstrate in humans due to continuous exposure with a number of pathogens, in comparison to inbred mice raised in a controlled laboratory environment. Furthermore, due to constant exposure to various pathogens, the memory T cell pool of an individual is also constantly changing. In an adult human, cross-reactive T cells represent a pool of cells ready to respond to a new pathogen. The quality and quantity of these cells are ultimately dependent upon an individual's immune history resulting from previous infections. T cell responses to a defined Hepatitis C virus (HCV) encoded HLA-A2-restricted non-structural protein 3 derived epitope NS31073–1081 was found to stimulate a cross-reactive T cell response to an Influenza virus encoded Neuraminidase antigen derived NA231–239 epitope in HCV-naive individuals (60–62). Similarly, our studies have demonstrated T cell responses against a number of HCV antigens in individuals who are otherwise HCV-naïve but are seropositive for Adenovirus (52). An earlier study also reported an abundance of pre-existing memory T cells against HCV NS3-1073 epitope from healthy HLA.A2 positive HCV-seronegative donors. Low dose exposure or acute clearance of HCV of the cohort was excluded in this study, and their origin from previous heterologous infections was suggested (63). Cross-reactivity of CD8^+^ T cells generated against influenza antigen with HCV NS3-1073 epitope was also demonstrated to result in severe liver pathology in 2 out of 8 acute HCV-infected patients (64). Broad cross-reactivities in T cell responses have been demonstrated between Epstein-Barr Virus (EBV) and Influenza virus epitopes (65). Further, despite broad cross-reactivities, it has been shown that selective CD8^+^ cross-reactive T cell repertoires against M1 antigen of influenza A virus and the early antigen BM of EBV play a significant role in disease severity of acute infectious mononucleosis during the acute EBV infection (66).

Infection with Dengue virus (DENV) in humans can sometimes lead to dengue hemorrhagic fever and shock syndrome. This severe immunopathology following DENV infection has been associated with re-exposure of individuals immune to one strain (serotype) of DENV with another strain. It has been demonstrated that cross-reactive non-neutralizing antibody can bind to viruses without inactivating them and enhance the infection of macrophages that bear Fc receptors for those antibodies (67). Furthermore, extensive T cell cross-reactivity occurs between different serotypes of DENV and a T cell response to the second dengue virus infection may induce CD8 T cells that have a higher affinity to the previously encountered Dengue virus, dampening the T cell response to the second virus. Therefore, both antibody-dependent immune enhancement and cross-reactive, low affinity T cell responses may combine to exacerbate the disease pathology (68).

## Natural Resistance to Infections in the Framework of Heterologous Immunity

**Figure 2:**
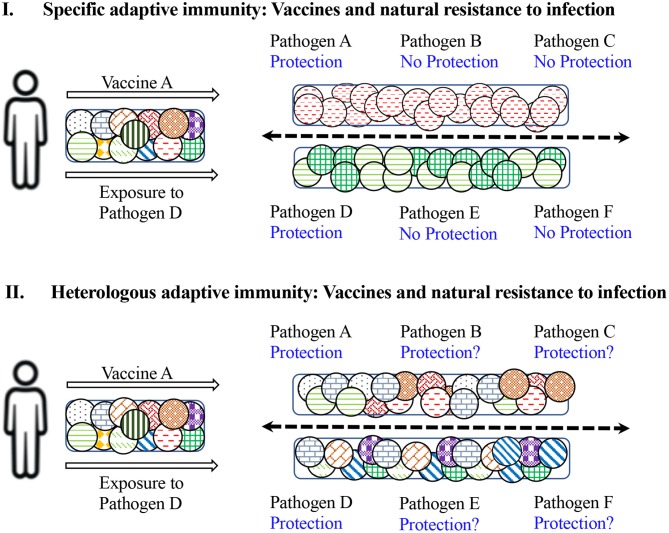
Impact of specific vs. heterologous adaptive immunity on natural and vaccine-induced resistance to infection. **(I)** Depicts the results of specific immunity. Upon administration with vaccine against pathogen A, a limited repertoire of naïve T cells specific to antigens of vaccine A will be induced and an individual would be protected against subsequent exposure with pathogen A, but not against pathogens B and C. Similarly, exposure or infection with pathogen D will stimulate and expand T cells specific against D and protect against re-infection with pathogen D but not against subsequent infections with pathogens E and F. **(II)** Demonstrates the consequences of heterologous immunity. Upon administration with vaccine against A, a broad repertoire of cross-reactive T cells will be activated and expanded, and an individual would be protected against subsequent exposure with pathogen A, but also protected against pathogens B and C to some extent. Similarly, exposure or infection with pathogen D will induce cross-reactive T cells and protect against re-infection with pathogen D, but may also protect against subsequent infection with pathogens E and F. Therefore, the exposure history of an individual may modulate the outcome of future infections with multiple pathogens.

Infection of humans with various pathogens such as Influenza virus, Respiratory Syncytial Virus (RSV), HIV, Hepatitis B Virus (HBV), HCV, DENV, Zika Virus (ZikaV), West Nile Virus (WNV), Poliovirus, and EBV leads to variable outcomes with respect to self-clearance, severe pathology, mortality and/or persistence of infection (69). Humans are not naïve to foreign antigens and pathogens. Previous exposure to pathogens leads to induction of innate and adaptive immune responses, which result in establishing a substantial pool of memory B and T cells long after the pathogen insult has been eliminated. These memory cells provide a fast and efficient protective response upon re-exposure to the same pathogen/antigen, fulfilling the specificity, and memory mandates of adaptive immunity (Figure 2). However, these also form a pool of ready-to-respond cross-reactive cells with low stimulation requirements. Demonstration of T cells reactive against various antigens of viruses such as HCV, HIV, Human Cytomegalovirus (HCMV), and herpesviruses, from individuals never-exposed or naïve to these pathogens, point to the existence of cross-reactive T cells (70–72). However, their precise contribution to success or failure of infection with these pathogens is still not clear (Figure 2).

As an example, infection with HCV presents an interesting scenario. There are currently ~70 million chronically infected patients worldwide (64) (https://www.who.int/news-room/fact-sheets/detail/hepatitis-c). In 70–85% of the exposed individuals, chronic persistent infection follows, whereas the remaining 15–30% successfully clear asymptomatic or symptomatic acute infection. Induction of immune responses has been shown to be delayed in infected individuals, but it is not clear if some pre-existing cross-reactive immunity plays a role in spontaneous clearance of an acute infection (73). An interesting clinical observation has been that super-infection with Hepatitis A Virus (HAV), Hepatitis B virus (HBV), and Hepatitis D virus (HDV) in HCV-positive patients is associated with decreased HCV-RNA replication or HCV clearance (74). Further, three lines of observations suggest the role of cross-reactive immunity in natural immunity/resistance to HCV: (1). T cells against various HCV antigens are found in the blood of individuals who are not HCV-positive, not at risk for being HCV-positive and are seronegative for HCV (62, 63, 70, 75), and our earlier studies showed induction of TH1 or TH2 types of T cell responses from HCV-naïve individuals in an ~1:2 ratio, which may reflect their propensity to develop acute vs. chronic infection upon exposure (76). (2). In seronegative individuals, HCV clearance has been reported after acute infection (60). (3). In some patients with persistent infection, spontaneous resolution of infection and seroconversion to antibody negative status has been found (77). In a group of blood donors with negative or indeterminate presence of anti-HCV antibodies and negative HCV RNA levels, a significant proportion had T cells that showed HCV antigen-specific T cell responses (78). Possible factors to explain this include (a) prior undetectable HCV exposure followed by clearance resulting in the induction of T cells reactive against HCV antigens, (b) abortive infection, or (c) occult infection with HCV. However, the possibility of cross-reactive T cells showing such responses cannot be excluded, especially since our studies have demonstrated robust cross-reactivity between commonly found adenoviruses (Ad5) and HCV (52, 79). The intriguing example of HCV demonstrates the complexity in determining the role of heterologous immunity in pathogenesis or protection from an infection, but undoubtedly indicates its significant role in natural immunity against a pathogen.

## Role of Microbiota in Heterologous Immunity

In addition to infectious pathogens, a host harbors a rich ecosystem of microbes in the order of 100 trillion microbes/person, consisting of Archaea, viruses, bacteria, fungi, protozoa and helminths. These microbiota are enriched at mucosal barriers such as gut and respiratory mucosa. Microbiota play an integral role in immune and metabolic homeostasis in an individual and can also contribute to control or prevent infection with a pathogen (80). Microbiota can either directly inhibit infection with pathogens by competing for an available niche, or indirectly inhibit infection through immune-mediated mechanisms (81). The microbiota can train/activate the innate immune system including antigen presenting cells, NK cells and innate lymphoid cells (ILCs), as well as induce a cross-reactive B and T cell repertoire able to recognize pathogens. Microbiota can also either allow the persistence of peripheral memory T cells or induction of regulatory T cells to induce tolerance to some pathogenic antigens. It has been suggested that the variety of antigens derived from the members of the microbiota can prime and lead to a diverse repertoire of memory T and B cells. These, in turn, can demonstrate enhanced immunity to a newly exposed pathogen through cross-reactivity (81).

Humans do not express the carbohydrate Galα1-3Galβ1-4GlcNAc-R (α-gal) and antibodies (both IgG and IgM) directed against α-gal are prevalent in human blood. Induction of α-gal-specific Abs is thought to be driven by exposure to bacterial components of the microbiota that express α-gal (82), such as *Klebsiella* spp., *Serratia* spp., and strains of *Escherichia coli*. Antibodies against α-gal have been shown to protect humans against the transmission of malaria (a protozoan *Plasmodium* infection), demonstrating the role of gut microbiota-induced specific acquired immunity that provides heterologous protection (83). Antibodies against α-gal have also been shown to be produced in humans infected with Gram-negative bacteria *Salmonella* spp. and protozoan parasites *Trypanosoma* spp. (84). It remains to be examined whether commensal- induced anti-α-gal antibodies also mediate heterologous protection against *Salmonella* spp. and *Trypanosoma* spp.

Microbiota-mediated induction of pre-existing antibodies against HIV gp-41 has been shown to be detrimental to the induction of neutralizing antibodies against HIV Env gp-120. This was seen in recipients of a DNA primed–rAd5 boosted HIV-1 vaccine in clinical trials (85) where a microbiota-mediated heterologous immune responses had a negative effect on vaccine efficacy.

## Vaccine Vectors in the Context of Heterologous Immunity

The most successful human vaccines so far have been live attenuated viral or bacterial pathogens such as Measles, BCG, Poliovirus (oral polio vaccine), and Smallpox virus. These vaccines tend to induce life-long immunity. The successes of these vaccines led to their application as delivery vectors for an unrelated antigen in their makeup. Subsequently, various attenuated bacteria such as *Escherichia coli, Vibrio cholerae*, lactic acid bacteria (LAB), BCG, *Listeria* spp., *Shigella* spp., *Salmonella* spp., and viruses such as Pox viruses, measles, modified vaccinia Ankara (MVA) and replication-deficient adenoviruses (Ad) have been tested for the targeted delivery of various antigens of bacterial, viral and parasitic origin into a variety of animal hosts (86).

Ad are commonly used as vectors to deliver transgenes in gene therapy and vaccines (87–89). Natural exposure to Ad is prevalent in the human population (up to 90% in some parts of the world based on antibody detection) and may lead to induction of neutralizing antibodies, which may reduce the generation of immunity against the transgene antigen (89). In addition to respiratory exposure, Ad have been shown to remain in the human gut for extended periods, making them akin to members of gut microbiota (90). Neutralizing antibodies against the vaccine vector has been the most studied response with respect to the immune response to Ad vectors, however it has also been suggested that by modifying the subtype/serotype, route and dose of Ad vector vaccines, one can circumvent the detrimental effect of pre-existing neutralizing antibodies. Consequently, rare human Ad and Ad of different animal species such as from cattle and chimpanzees have also been tested as vaccine vectors (91, 92). Although it has been shown that neutralizing antibodies don't cross-recognize Ad of different serotype or species, T cell cross-reactivity between different Ad has been demonstrated in humans (93). It has been shown that in contrast to B cell epitopes, common T cell epitopes are present in conserved regions (~80%) of the Ad hexon protein. Due to the presence of conserved T cell epitopes in hexons, cross-reactivity among divergent serotypes from chimpanzees and humans has been observed. It has been shown that Ad-specific T cells are universal in humans even in low prevalence areas of the world, although the magnitude of CD4^+^ and CD8^+^ T cell response may vary among individuals. Further, it has been suggested that Ad-specific CD8^+^ T cells remain in an effector-memory-like state and can readily and rapidly perform effector functions upon re-stimulation. By virtue of T cells being cross-reactive, these pools of Ad-specific T cells that are present universally in humans may provide an efficient source of effector T cells to target heterologous pathogens that regularly infect people.

Our studies have demonstrated unexpected and surprising homologies between peptides of HCV antigens and Ad antigens, and robust cross-reactive cellular and humoral immunity between Ad and HCV (52). It remains to be investigated whether Ad-specific effector-memory T cells provide the immediate defense when exposed to infecting HCV, and whether they form at least a partial basis for the 15–30% spontaneous clearance of HCV observed in humans. In a follow up report, we demonstrated that cross-reactive cellular and humoral immune responses against HCV antigens core, NS3, and NS5 are also induced upon immunization with various recombinant Ads containing antigens from HCV, *Mycobacterium tuberculosis*, HIV, and EBOV (94). Intriguingly, the nature of the transgene antigen had a significant impact on the levels of cross-reactive immunity induced against HCV antigens (94). It is possible that Ad also has cross-reactivity with other pathogens, but this has not been explored yet. These observations shed light on another rather unstudied aspect of vaccine vectors and support the notion that heterologous immunity induced by vaccine vectors may lead to significant heterologous immunity against another pathogen possibly influencing the natural course of infection with that pathogen. Consequently, non-specific effects of vaccine vectors must also be examined thoroughly.

## Discussion and Future Prospects

Specificity and memory are the two traits of the adaptive immune system exploited for the development and application of vaccines. It is expected that administration of a vaccine would induce vaccine-antigen-specific T and B cell memory responses, which upon exposure to the corresponding pathogen, would rapidly and specifically lead to prevention or clearance of the infection. Many prophylactic and therapeutic vaccines, experimental or clinical, include antigens in various forms from pathogens, tumors, allergens, and/or autoantigens, and are designed to employ specific adaptive immunity, supported by adjuvants which mobilize the collaboration of non-specific, innate immunity. Experimental and clinical evidence as well as theoretical constructs have clearly demonstrated that heterologous immunity or cross-reactivity of adaptive immune cells is not an isolated or accidental phenomenon, but rather a fundamental attribute of adaptive immunity, forming an integral part of the host defense system against pathogens under natural conditions. Application of heterologous immunity in vaccines can be contemplated in chronic infections and cancer, where antigen-specific lymphocytes have become anergic and/or exhausted and don't respond to a specific vaccine. Mobilizing cross-reactive adaptive immunity by a heterologous therapeutic vaccine may be an ultimate strategy to induce effective protective immunity in such chronic disease conditions. Furthermore, as stated earlier, cross-reactive lymphocytes may not be the only mechanism behind heterologous immunity, and trained innate immunity could explain some of the observations of heterologous immunity. Therefore, it should also be pursued in future vaccine design and efficacy studies.

In an individual, T and B cell repertoires originate by random VDJ gene rearrangement, however, their cross-reactivity and ability to respond to various antigens in a host is shaped to a large extent by exposure history and microbiota, and therefore, cross-reactivity remains largely unpredictable at the individual level. Acknowledgment of the heterologous side of adaptive immunity does have important consequences on self-tolerance, autoimmunity, and vaccination strategies. Potential positive heterologous effects of vaccines have been discussed in this article, but heterologous immunity can also have dire consequences upon cross-reacting to self-antigens. It is essential that future investigation of vaccine design must exploit the beneficial aspects of heterologous immunity and at the same time devise strategies to avoid the potentially harmful effects. As stated earlier, cross-reactivity may vary among individuals and different sexes, and may largely remain unpredictable making application to vaccines which are usually population-based, not an easy task. In addition, recent understanding of the role of microbiota in immune homeostasis and induction of immune tolerance in a host has opened new avenues of investigation in vaccines. Specifically, it has been shown that pathological precipitation of many of the autoimmune diseases e.g., multiple sclerosis, type 1 diabetes, rheumatoid arthritis and systemic lupus erythematosus, are controlled to a large extent by environment and microbiota (95, 96). Moreover, even in individuals with genetic susceptibility and the peripheral presence of potentially autoreactive T cells, modifications of gut microbiota may allow modulating the disease pathology (97). Therefore, it can be inferred that self-reactive (autoimmune) consequences of vaccines may be prevented/circumvented through modulation of gut microbiota. Additionally, development of tools and techniques to predict cross-reactivity against both unrelated pathogens and self-antigens would aid in vaccine design and coverage of protection obtained by a vaccine.

## Author Contributions

BA collected the literature, wrote, and revised the manuscript.

### Conflict of Interest

BA is coinventor on a PCT application relating to heterologous immunity between adenoviruses and Hepatitis C virus.
